# Association of Parent and Child Intuitive Eating: A Scoping Review

**DOI:** 10.1177/15598276241279223

**Published:** 2024-09-06

**Authors:** Michaela L. Dowling, Madeline E. Hubbard, Richa Agnihotri

**Affiliations:** 1 Department of Medicine, 3710 McMaster University, Hamilton, ON, Canada (MLD, MEH); 2 Faculty of Health Sciences, 3710 McMaster University, Hamilton, ON, Canada (RA)

**Keywords:** parent, child, intuitive eating, review

## Abstract

Children’s eating behaviors are dependent on childhood food experiences, which involve their parental feeding practices, home food environments, and modeling of eating behavior. Intuitive eating (IE) promotes eating based on internal hunger and satiety cues. IE has been associated with improvements in mental and physical health. There has been increasing interest in exploring the association between parent and child IE. The aim of this scoping review was to synthesize current literature reporting on parent and child IE associations. Four databases (MEDLINE, EMBASE, Web of Science and CINAHL) were searched using keywords focusing on IE, parents, and children. Inclusion criteria were reporting on parental and/or child IE, and reporting on parent–child relationships. After screening, 15 studies were retained. From these, 3 main correlations were described. Parental IE was associated with child feeding, child weight concerns, and the home food environment. As well, environmental factors (i.e., family cohesion, food security) were associated with components of child IE. Moreover, IE was directly correlated between parents and children. Overall, this study highlights how child IE behaviors may be shaped by both parental IE and the broader environments that they are raised within. Additional high-quality studies are required to verify these findings.

“All of family cohesion, encouraging diet diversity, healthy eating guidance, and positive parental coping mechanism/low parental stress were positively associated with child IE.”

## Introduction

The desire to eat can be traced back to the influence of motivators such as emotions, external contexts (i.e., food sight or odor), or physical hunger cues.^1^ People who practice intuitive eating (IE), which involves eating based on physiological hunger and satiety cues, experience numerous health benefits.^2^ IE describes a weight-inclusive eating pattern subdivided into four primary eating behaviors: unconditional permission to eat (UPE), eating for physical rather than emotional reasons (EPR), reliance on hunger and satiety cues (RHSC), and body-food choice congruence (B-FCC).^3^ Adults who adhere to IE have a variety of adaptive benefits including increased self-efficacy, self-esteem, optimism, and life satisfaction.^1,4-6^ IE is also associated with a decreased risk of disordered eating and body dysmorphia.^4^ IE interventions for eating disorders have led to a decrease in the number of individuals meeting diagnostic criteria for eating disorders and a decrease in binge eating frequency.^3^

The ability to regulate one’s hunger based on internal signals is innate to human behavior. This enables young children to attain the proper nutritional intake to facilitate their development.^7^ As children age, their eating behaviors become heavily influenced by childhood food experiences, which include their parental feeding practices, home food environments, and parental modeling of eating behavior.^7^ Children can develop maladaptive eating patterns through the influence of each of these factors, which predisposes them to the development of physical and mental ailments.

Parental feeding practices have been evaluated with two main modalities: the Satter Division of Responsibility (sDOR) and the Child Feeding Questionnaire (CFQ). The sDOR stresses the importance of the parent-child relationship and the interplay between their respective responsibilities as the feeder and the eater.^8^ Within this model, the child’s eating behavior is described as a learned process that is actively shaped by parental feeding practices.^8^ In contrast, the CFQ describes parental feeding practices as methods used to correct child eating behaviors that are considered inappropriate. Although both models have produced valuable insight into parental feeding practices, the current study will focus on the latter.

The CFQ describes the feeding behaviors parents exhibit to obtain a desired eating behavior from their child.^9^ Each of the three subscales of the CFQ (restriction, pressure to eat, and monitoring^10^) have been associated with different eating behaviors in children. Restrictive feeding has been associated with uninhibited eating and excessive weight gain in children.^11,12^ Parental pressure to eat has been associated with lower fruit and vegetable consumption, higher fat intake, and lower responsiveness to internal hunger cues in children.^13-16^ In particular, pressure to eat was associated with dietary restraint in young girls, which has implications for the development of eating disorders.^16,17^ In contrast, parental monitoring of their child’s food intake was protective against emotional eating in children.^18^ Overall, there is substantial evidence that parental feeding practices influence both the diet quality and eating patterns of their children.

Children’s dietary habits are also influenced by the food environment in which they are brought up. Those with greater access to fruits and vegetables have both a greater intake in their diet and a greater preference for healthy foods.^19-3^ As parents and guardians largely determine the home food landscape, their dietary patterns influence those of their children. Children’s intake is directly correlated with the amount of food presented in front of them. Children offered larger meal portions consume more energy than those offered smaller portions.^24^ As parents portion meals based on the amount of food that they personally consume, children’s food intake is indirectly related to parental eating behaviors.^25^

It is well-established that children learn via social modeling which involves learning a behavior through the observation of another individual or model. Children’s eating behaviors are modeled after the behavioral patterns of their parental figures. Parents who model healthy diets, such as consuming fruits and vegetables, have children who abide by similar diets.^22,23,26-30^ Mothers who practice eating behaviors such as emotional eating, eating for external reasons, or restrained eating are more likely to have children who demonstrate the same eating behaviors.^31^ If parents model IE, their children may also participate in this eating style.

Parents and guardians aim to provide children with the resources, knowledge, and skills to develop into healthy adults. Promoting IE could lead to adaptive benefits for children. To promote IE in children, it is important to understand the factors that facilitate this eating behavior. There has been increasing interest in exploring the association between parental and child IE. While it is known that parental feeding, home food environments and social modeling influence children’s eating patterns, the specific relationships between parental and child IE have yet to be summarized in a review. The aim of this scoping review was to amalgamate the current literature regarding the influences of parental IE on their children, as well as parental factors that contribute to children’s IE.

## Methods

The PRISMA-ScR protocol for scoping reviews was followed and the reporting guideline for the manuscript was the PRISMA Extension for Scoping Reviews.^32^ The protocol was registered on Open Science Framework (OSF: https://osf.io/uk36b).

### Eligibility Criteria

In this scoping review, literature was included that reported on the association between parent and child IE. More specifically, included literature either reported the effect of parental IE on children, or reported parental influences of children’s IE. Inclusion criteria were reporting on parental and/or child IE using a validated model of IE, and reporting on parent–child relationships. No age restriction for children was included to ensure that both prospective studies and retrospective studies reporting on childhood food experiences were included. Inclusion criteria were publication in English in a peer-reviewed journal. Studies were not restricted based on date, geographical location or study design. Grey literature and unpublished literature were excluded.

### Information Sources and Search Strategy

After a preliminary review of current literature on IE for parents and children, a search strategy was developed using keywords focusing on IE, parents and children. An example search of MEDLINE is provided in the Supplemental Material (Table S1). The databases included in the search were MEDLINE, EMBASE, Web of Science and CINAHL.

### Study Screening

Literature retrieved through searching databases was uploaded to Covidence. After removing duplicates, 2 reviewers screened studies based on their titles and abstracts. To ensure a common understanding of eligibility criteria, a pilot trial was run with both reviewers assessing ten studies together. Afterward, both reviewers independently screened all remaining studies. Full-text screening followed the same process with a pilot trial of 5 studies.

### Data Extraction

An Excel spreadsheet was used to extract data from the included studies. Two reviewers extracted data from 2 studies together. Then, they each extracted data from half the remaining studies. Both reviewers verified the data extraction of the other reviewer. Specific data extracted included demographics of parents and children, parents’ eating or feeding behavior, children’s eating behavior, the effects of parental IE on children, parental influences on children’s IE, and other outcomes related to parents or children (e.g., mental health, etc.). Both patient-reported and observer-reported outcomes were presented. Missing data was noted.

### Synthesis and Presentation of Results

Data was summarized individually and synthesized. First, associations between parent IE and child feeding behaviors and home food environment were summarized. Then, parental influences on children’s IE were described. If sufficient data was retrieved, a synthesis of results of IE interventions for parents or families would be presented.

## Results

### Summary

**Figure 1.**
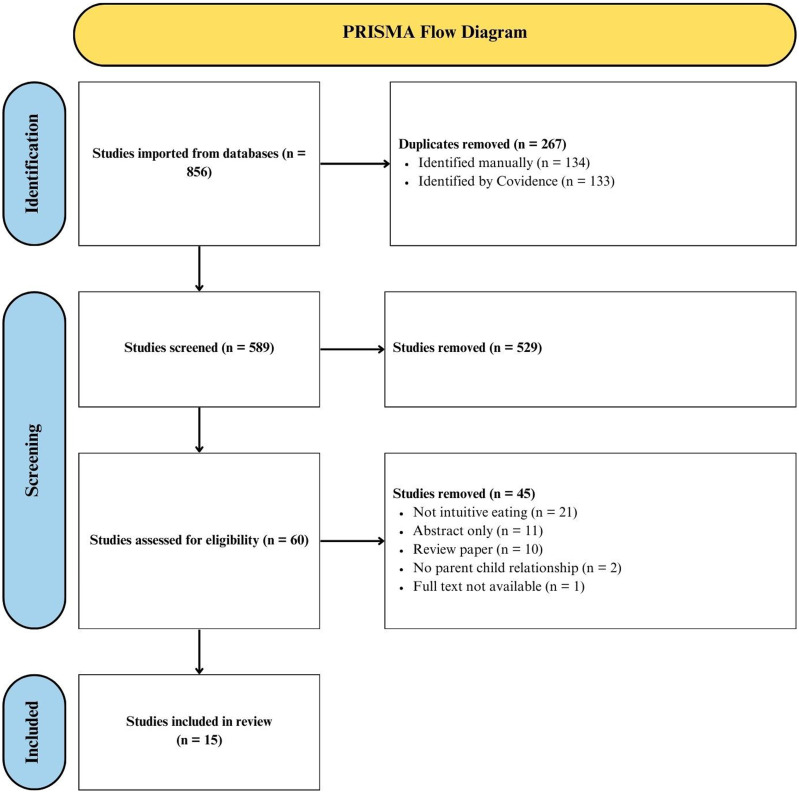
PRISMA flow diagram.

Through the aforementioned selection process, 15 papers were retained for further analysis and data extraction. A PRISMA flow diagram depicting study selection is presented (Figure 1). Of the 15 identified studies, 2 were population-based cross-sectional longitudinal studies,^33,34^ 6 were cross-sectional studies,^35-40^ 6 were retrospective cross-sectional studies,^41-46^ and 1 was a retrospective chart review.^47^ Included studies were published from 2010 to 2024 with most (n = 9) of the studies being completed during or after 2020.^33-35,37,40,43,45-47^ A total of 11 studies were completed in the United States,^33-39,42-44,47^ while 1 study was completed in each of Canada,^46^ China,^40^ and Poland.^45^ The last study involved a portion of its sample from both the United States and United Kingdom.^41^ IE was reported using the full 21-item Intuitive Eating Scale (IES^4^) by 5 studies,^38,39,41,42,44^ the full 23-item Intuitive Eating Scale 2 (IES-2^47^) by 6 studies,^36,40,43,45-47^ a modified version of IES by 3 studies,^33-35^ and a modified version of IES-2 by 1 study.^37^ A few of these papers reported on outcomes unrelated to IE. These outcomes were excluded for the purpose of this report. Findings pertaining to IE are highlighted below.

### Effect of Parental IE on Child Feeding Behaviors and Food Environment

Five studies investigated the influence of parental IE on child feeding behaviors and food environment (Table 1).^36-39,47^ In total, the studies analyzed the responses of 1358 parents of which 1045 (76.95%) identified as mothers and 313 (23.05%) as fathers. Child ages ranged from 8.8 ± 2.0 months,^36^ to 15.4 ± 1.43 years.^47^ Overall, both total parent IE scores and the subcategories of IE were associated with the food environment and feeding patterns of children.

**Table 1.** The Impact of Parental IES on Child Feeding Behaviors, Food Environment, and Eating Patterns.

			Parental Intuitive Eating				
Study	Parent Characteristics	Child Characteristics	Total	UPE	EPR	RHSC	B-FCC
Khalsa et al, 2019	N = 201	Age: 8.8 ± 2.0 mo	/	Neg: Restrictive feeding style	/	Pos: Responsive feeding style	Pos: Restrictive feeding styles
	Age: 27.2 ± 5.7	Female: 50%					
	Mother: 90%	Male: 50%		Pos: Laissez-faire feeding style and indulgent feeding style			
	Father: 9.5%	BMI z score 0.35 ± 1.08					
Nelson et al, 2023	N = 47	N = 47	/	/	Pos: Weight gain in eating disorder treatment	Neg: Adolescent weight concern, shape concern, and eating disorder behavior	Neg: Adolescent weight concern, shape concern, and eating disorder behavior
	Mother: 72.3%	Age: 15.4 ± 1.43					
	Father: 10.6%	Female: 87.2%					
	Other: 2.4%	Male: 12.8%					
		BMI: 18.9 ± 2.3					
Rodgers et al, 2022	N = 750	Age reported by mothers: 5.1 ± 3.4	Neg: Salty snacks and soda pop in home (mothers)	/	/	/	/
	Mother age: 31.3 ± 1.5						
	Father age: 31.5 ± 1.4	Age reported by fathers: 4.0 ± 2.9	Pos: Availability of fruits and vegetables in the home (mothers), fruit served at meals (mothers), and vegetables served at dinner (both)				
	Mother: 62.7%						
	Father: 37.3%						
Tylka & Kroon Van Diest, 2013^48^	N = 180	Age: 3.40 ± 0.98	/	Neg: Restrictive feeding	Pos: Monitoring child food intake	/	/
	Age: 34.31 ± 6.05						
	Mothers: 100%						
	BMI: 26.41 ± 6.74						
Tylka et al., 2015	N = 180	Age: 3.40 ± 0.98	/	No influence on relationship between child weight concern and restrictive feeding	Child weight concern Pos. to restrictive feeding parents with low or average EPR, no association parents with high EPR	Child weight concern Pos. to restrictive feeding parents with low or average RHSC, no association parents with high RHSC	/
	Age: 34.31 ± 6.05						
	Mothers: 100%						
	BMI: 26.41 ± 6.74						

IES-2 = intuitive eating scale; UPE = unconditional permission to eat; EPR = eating for physical rather than emotional reasons; RHSC = reliance on hunger and satiety cues; B-FCC = body-food choice congruence; Neg = negative association; Pos = positive association.

Three studies specifically analyzed the influence of parental IE subscales on child feeding practices.^36,38,39^ Two studies found a negative correlation between parental UPE scores and the use of a restrictive feeding style.^36,38^ Additionally, the study by Tylka, Lumeng and Eneli found that EPR and RHSC moderated the interaction between parental concern about their child’s weight and restrictive feeding.^39^ Indeed, in parents with high levels of EPR and RHSC, weight concern and restrictive feeding were unrelated. However, in parents with low or average EPR and RHSC, there was an association between concern about their child’s weight and restrictive feeding.^39^ In contrast, restrictive feeding styles were positively correlated with parental B-FCC.^36^ In addition to these findings, different parental IE behaviors increased the prevalence of other feeding behaviors. For instance, UPE was positively correlated to both laissez-faire and indulgent feeding styles, and RHSC was directly correlated to a responsive feeding style.^36^ There was also a positive association between EPR and monitoring a child’s food intake.^38^ Altogether, the different subcategories of parental IE have differing impacts on feeding behaviors.

The work by Rodgers and colleagues^37^ examined the relationship between parental total IES-2 scores and the food environment in the home. They demonstrated that maternal IE was associated with many aspects of home food availability and meal experiences. In particular, maternal IE was negatively correlated with the availability of salty foods and soft drinks in the house, and positively correlated to the availability of fruits and vegetables in the home and serving them with meals. Although paternal IE was not associated with the home food environment, they found that fathers with higher IE tended to serve more vegetables at dinner. Together these findings suggest that parental total IE facilitates a healthier food environment.

Nelson and colleagues^47^ assessed parental IE as it pertains to facilitating their child’s weight restoration during eating disorder treatment. From this research, they demonstrated that both parental RHSC and B-FCC were negatively correlated to the child’s eating disorder behavior, weight concern, and shape concern. Moreover, a higher parent EPR was associated with increased child beneficial weight gain following the treatment regimen. This finding indicates that parental IE may have the potential to foster healthy eating behaviors in children with eating disorders to uphold their physical health.

### Parental Factors Contributing to Child Total IE and IE Subscales

Ten papers reported on parental influences of children’s intuitive eating (Table 2).^33-35,40-46^ Altogether, these papers include 6766 children and 3631 parents. Five studies did not report on the number of parents included or demographic characteristics.^33,40,43-45^ Two studies collected data from adolescents.^33,34^ All ten studies included retrospective reports from adults of childhood influences on eating behavior. Of the children participants, 3843 (56.8%) were female, 2910 (43.0%) were male, 10 (0.1%) identified as another gender, and 3 (0.01%) were unreported. A total of 2386 (65.7%) of parents identified as female/mothers and 1009 (27.8%) identified as males/fathers. The rest of the parents identified as another parental figure or were unreported. Overall, children’s total IE and IE subscales were influenced by several parental factors.

**Table 2.** Parental Factors Contributing to Child Total IES and IES Subscales.

			Child Intuitive Eating				
	Parent Characteristics	Child Characteristics	Total	UPE	EPR	RHSC	B-FCC
Burnette et al, 2023 “Is intuitive eating…”	Not reported	N = 1372	Neg: Food insecurity in adolescence and early adulthood	/	/	/	/
		Age: 14.4 ± 2.0 (baseline); 22.0 ± 2.0 (follow-up)					
		Female: 53.1%					
		Male: 46.9%					
Burnette et al, 2023 “How parental feeding…”	N = 2136	N = 1383	Neg: Restrictive feeding, especially with low parental weight concern	/	/	/	/
	Female age: 41.6 ± 7.8	Age: 14.4 ± 2.0 (baseline); 22.0 ± 2.0 (follow-up)	Neg: Pressure to eat (adolescent males; emerging adult at low parental weight concern)				
	Male age: 44.8 ± 8.4	Female: 52.7%	Pos: Pressure to eat (adolescent females; emerging adult at high parental weight concern)				
	Female: 60.6%	Male: 47.3%					
	Male: 39.4%						
Burnette et al, 2022	N = 891	N = 891	Neg: Perceiving the child to be overweight (parent and child)	/	/	/	/
	Age: 50.4 ± 8.0	Age: Other: 0.7%22.0 ± 2.0	Pos: Perceiving weight to be “about right”				
	Mother: 74.2% father: 18.4%	Female: 53.1% male: 45.8%					
Ellis et al, 2016	N = 170	N = 170	Neg: Pressure to eat during childhood (female students with higher BMI)	/	/	/	/
	Age: 48.26 ± 5.87	Age: 19.75 ± 1.99					
	BMI: 27.71 ± 8.87	Female: 71.2%					
		Male: 28.8%					
		BMI: 23.95 ± 4.66					
Galloway et al, 2010	N = 98	N = 98	/	/	Neg: parental monitoring of eating (especially female children)	/	/
	Age: 38-58	Age: 17-23			Neg: Restrictive feeding (female children only)		
	Mother: 97%	Female: 72.4%					
		Male: 27.6%					
Ge et al, 2024	Primary caregiver = Mother: 94.7%	N = 941	Pos: Healthful foods at home	Neg: Healthful foods at home (male children only)	Pos: Healthful foods at home	Pos: Healthful foods at home (female children only)	Pos: Healthful foods at home
	Father: 53.7%	Age: 44.35 ± 13.15	Pos: Encouraging diet diversity (female children only)	Pos: Honoring children’s hunger and satiety feelings (male children only)	Pos: Encouraging diet diversity (female children only)	Pos: Encouraging diet diversity	Pos: Encouraging diet diversity (female children only)
		Female: 53.2%	Pos: Honoring children’s hunger and satiety feelings (male children only)			Pos: Honoring children’s hunger and satiety (male children only)	
		Male: 46.4%				Pos: Considering child’s taste preference (male children only)	
		Other: 0.4%					
Kroon Van Diest & Tylka, 2010	Not reported	N = 238	Neg: Restrictive/critical eating messages	/	/	/	/
		Age: 20.87 ± 3.92					
		Female: 67.2%					
		Male: 32.8%					
Malachowska & Jezewska-Zychowicz, 2023	Not reported	N = 708	/	Neg: Restrictive feeding style	Neg: Restrictive feeding style (especially female children)	Neg: Restrictive feeding style (especially female children)	/
		Age: 36.9 ± 11.5			Neg: Pressure, food reward	Pos: Healthy eating guidance	
		Female: 67.4%			Pos: Healthy eating guidance		
		Male: 32.6%			Pos: child control of feeding (male children only)		
Roberts et al, 2020	N = 336	N = 263	Neg: Maternal BMI (female children only)	/	/	/	/
	Age: 56.62 ± 8.81	Age: 29.13 ± 6.65	None: Feeding practices (restriction, monitoring, etc.)				
	Mother: 100%	Female: 100%					
	BMI: 26.86 ± 5.30	BMI: 24.89 ± 5.30					
Yang et al, 2023	Not reported	N = 702	Pos: Family cohesion and positive coping styles (mediated by perceived stress)	/	/	/	/
		Age: 21.12 ± 1.48					
		Female: 45.4%					
		Male: 54.6%					
		BMI: 20.49 ± 2.69					

IES-2 = intuitive eating scale; UPE = unconditional permission to eat; EPR = eating for physical rather than emotional reasons; RHSC = reliance on hunger and satiety cues; B-FCC = body-food choice congruence; Neg = negative association; Pos = positive association.

Familial factors negatively associated with children’s total IE were parent and child perceptions that the child was overweight,^35^ and maternal body mass index (BMI) for female children.^46^ As well, food insecurity in childhood was associated with lower IE in adolescence and early adulthood.^33^ The availability of healthful foods at home was negatively associated with the subscale of UPE for male children.^43^ Finally, parental monitoring of eating was negatively associated with the subscale of EPR.^42^

Four papers found a negative association between parental restrictive feeding and children’s IE in one or more subscales.^34,42,44,45^ Restrictive feeding was associated with lower total IE score,^34,44^ especially when parents were not concerned about their children’s weight.^34^ Restrictive feeding was negatively associated with the subscale of EPR, especially in female children.^42,45^ Additional subscales negatively associated with restrictive feeding were UPE and RHSC.^45^ One paper found no association between restrictive feeding and children’s total IE scores.^46^

Three studies found a negative association between parental pressure to eat and children’s IE. One study found that pressure to eat was negatively associated with total IE scores in females with higher BMI.^41^ Another found a negative association between food reward and the subscale of children’s EPR. Total IE scores were negatively associated with pressure to eat in both adolescent males and emerging adults whose parents were not concerned about their weight.^34^ Contrarily, 1 study found that pressure to eat was positively associated with IE in adolescent females and emerging adults whose parents had high concern about their weight.^34^

Other factors positively associated with children’s total IE scores were perceiving the child’s weight to be adequate,^35^ family cohesion and positive coping styles,^40^ and the availability of healthful foods at home.^43^ Additionally, encouraging diet diversity and honoring children’s hunger and satiety were positively associated with total IE for females and male children, respectively.^43^ The availability of healthful foods at home, encouraging diet diversity, and healthy eating guidance were generally positively associated with subscales of IE including EPR, RHSC, and B-FCC.^43,45^

### The Association Parent IE & Child IE Behaviors

Two studies analyzed the direct association between parent and child IE.^35,47^ Nelson and colleagues^47^ found that parent RHSC and child total IE were positively associated. Moreover, parent and child B-FCC were positively correlated. Similarly, Burnette and colleagues^35^ found that 52.9% of parent–child dyads had concordant eating behaviors. Specifically, 31.2% of dyads were both IE, while 21.7% were both non-IE. The smallest category was of dyads wherein the child engaged in IE but the parent did not. Altogether, these findings illustrate direct associations between parent and child IE.

## Discussion

**Figure 2.**
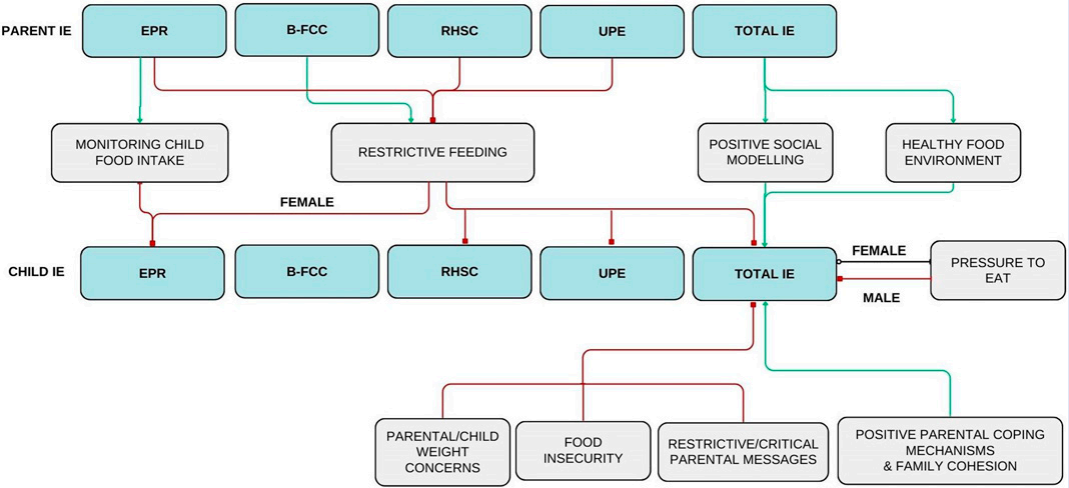
The direct and indirect pathways in parental facilitation of child intuitive eating. After analysis of the 15 retained studies, correlations between parental IE and child IE were extracted and variables mediating child IE were identified (gray boxes). The described variables were found to have a positive (green line) or negative (red line) association with a child’s tendency to exhibit IE behaviors. Some correlations only pertain to male or female children and are designated as such. The correlation between pressuring a female child to eat and said child’s total IE was conflicting in the literature; consequently, it is denoted with a black line. IE = intuitive eating; UPE = unconditional permission to eat; EPR = eating for physical rather than emotional reasons; RHSC = reliance on hunger and satiety cues; B-FCC = body-food choice congruence.

The current review evaluated the association between parent and child IE. The results suggest that parental IE may both directly and indirectly facilitate IE in children. This study found associations between numerous parental behaviors and child IE. These results confirm that parents have a substantial influence on the development of eating behaviors in their children. A summary of connections between parental and child IE is presented (Figure 2).

Parental IE indirectly facilitates child IE through three main variables: parental feeding practices, weight concerns for their children, and the home food environment. First, high parental IE scores, specifically in the subdomain of UPE, were negatively associated with restrictive feeding practices.^36,38^ As well, parental use of restrictive feeding decreased child IE behaviors.^34,42,44,45^ Previous literature has established a link between restrictive feeding practices and both uninhibited eating and excessive weight gain in children.^11,12^ From the results of this study, a model can be proposed wherein parents who do not engage in IE may be more inclined to turn to restrictive feeding than those who do. Subsequently, this may lead to a lower probability that their child will practice IE.

High parental EPR scores were positively associated with monitoring their child’s food intake.^38^ Interestingly, this feeding practice was correlated with a decrease in daughter’s EPR score.^42^ This finding contradicts a previous study which found that parental monitoring was linked to lower emotional eating in children.^18^ Perhaps this finding is related to children’s reliance on their guardians to select the timing and contents of their meals. Although monitoring did not encourage child IE, it does have beneficial implications on the child’s diet. Indeed, a previous study found that parental monitoring increased the chances that children would eat the recommended daily amounts of fruits, grains, dairy, and vegetables.^49^

The second manner by which parental IE is indirectly associated with child IE is through child weight concerns. As per the study completed by Tylka, Lumeng and Eneli, it was found that parental EPR and RHSC moderated the association between parental concern about their child’s weight and the use of restrictive feeding.^39^ Parents with higher IE scores in EPR and RHSC were less likely to use restrictive feeding in response to concern over their child’s weight.^39^ Moreover, Burnette and colleagues^35^ explained a negative correlation between child weight concerns and child total IE. It can be proposed that parents with higher IE are less likely to implement restrictive feeding in the context of concern for their child’s weight, which therefore promotes the development of IE behaviors in their child.

Parental IE may also increase child IE by influencing the food landscape of the home. Parents with higher total IE scores were more likely to have fruits and vegetables available in the household and less likely to bring salty snacks or soda into the home.^37^ When healthy foods were accessible to children, they were more likely to engage in IE.^43^ These two findings link parental IE to an increase in child IE. Additionally, other literature has reported that children with greater accessibility to healthy foods (i.e., fruits, vegetables, dairy) are more likely to develop a preference for said foods and incorporate them more frequently into their diet.^19-23^ Therefore, not only does parental IE promote children’s health by increasing their likelihood of practicing IE, but it also supports a well-rounded nutritious diet as well.

The current review also highlighted several parent-involved factors that influence child IE. All of family cohesion, encouraging diet diversity, healthy eating guidance, and positive parental coping mechanism/low parental stress were positively associated with child IE. Both food insecurity and critical parental messages directed to their child were negatively correlated with child IE. Of note, the implications of parental pressure to eat were conflicting. While two studies reported that pressure to eat was negatively correlated with IE in sons, the literature was not consistent for daughters.^34,45^ Two studies reported that pressure to eat was linked to decreased IE behaviors in daughters,^41,45^ whereas another study reported the opposite to be true.^34^ This finding is echoed in the broader literature that describes diverse outcomes of pressure to eat. Pressuring a child to eat has been associated with a lower consumption of fruits and vegetables, more snacking, and a higher fat intake.^3,13,14,16,17^ As well, pressure to eat has been associated with disordered eating behaviors in daughters including dietary restraint.^17^ Other studies have indicated the opposite, describing a decrease in snacking behavior and healthy eating patterns with pressure to eat.^50^ As such, the implications of pressuring a child to eat on their tendency to engage in IE and on eating behaviors in general requires further investigations.

Two studies demonstrated direct associations between parent and child IE.^35,47^ In particular, high parental scores of RHSC and B-FCC were positively associated with total child IE scores and child B-FCC scores, respectively.^47^ These findings are consistent with the well-established theory of social modeling. Previous literature has demonstrated that children who observe their parents select nutritious food are more likely to do so themselves, and that children often mimic the eating behaviors practiced by their parents.^22,23,26-31^

The results of this review could be applied in the development of family-based interventions aimed at increasing the overall health and well-being of children and youth. Past literature has shown IE to be an effective method of upholding an individual’s health.^2^ Directly promoting IE to youth via education or reinforcement may improve their future health outcomes. Interventions should simultaneously focus on the behaviors of the parents and guardians of these children. Education should be given to families on the importance of maintaining a healthy home food environment, the benefits/drawbacks of the different feeding practices, and the importance of social modeling. As well, interventions should look at each family unit holistically and consider addressing underlying causes of food insecurity or family unrest that may impede children from developing IE habits.

The importance of lifestyle medicine, especially with regards to the pediatric population, is a rapidly developing area of interest. To date, there are no known studies which synthesize the literature pertaining to the effects of parental IE on their children. Instead, other authors have focused on the effects of other parental eating behaviors such as eating disorders or mindful eating/parenting.^7,51,52^ This is likely due to the novelty of research on IE, which is reflected in the fact that most of the literature included in this report was published from 2020 to 2024. This review summarizes current literature to provide novel insight into the associations of specific subdomains of IE (i.e., UPE, EPR, RHSC, and B-FCC) between parents and children. Specific parental factors can be targeted to facilitate the development of child IE.

A limitation of this study is that, due to the novelty of research in this field, only 15 studies were included in this scoping review. As well, studies were included regardless of children’s age. This could complicate data interpretation as children become more independent in their feeding choices as they age. Studies were only included if they clearly demonstrated a relationship between IE amongst parents and children. However, there may be other parentally influenced variables that have yet to be directly correlated to child IE. For instance, higher screen use both during and outside mealtimes results in lower IE in the adult population.^53,54^ Correspondingly, children who spend more time on screens have poorer dietary patterns^55-58^; however, published literature has not described the influence of screen time specifically on child IE. As primary caregivers greatly influence their children’s screen time,^59^ this may be another means by which parents influence their child’s IE behaviors. Therefore, it is plausible that other variables were not described in this review due to the lack of direct correlations in the current literature.

Additionally, none of the studies referenced in this review were randomized control trials or interventional studies, rather the results concluded here were from retrospective reports and cross-sectional analyses. Consequently, causative conclusions cannot be drawn from the work currently available. It is also important to note that 11 of the 15 studies were published in the United States, so it is possible that the associations depicted in this paper may only be applicable to certain geographical locations. Further research in different countries is required to verify the results of the current review.

Nonetheless, the findings from the current review suggest that parental IE directly increases the likelihood of children developing IE. Parental IE may also indirectly increase child IE through the pathways of parental feeding practices, and the home food environment. Moreover, additional factors such as food security, family cohesion, parental weight concern, and feeding practices all impact eating behaviors in children. Additional high-quality studies are required to verify these findings.

## Supplemental Material

Supplemental Material - Association of Parent and Child Intuitive Eating: A Scoping Review Supplemental Material for Association of Parent and Child Intuitive Eating: A Scoping Review by Michaela L. Dowling, Madeline E. Hubbard, and Richa Agnihotri in American Journal of Lifestyle Medicine

## Data Availability

The raw data extraction table is available upon request to the corresponding author.*
