# Supplementary material for: Association of Parent and Child Intuitive Eating: A Scoping Review
Source: Am J Lifestyle Med. 2024 Sep 6:15598276241279223. Online ahead of print. doi: 10.1177/15598276241279223 (PMC11562447; doi:10.1177/15598276241279223)
Supplement: Supplemental Material - Association of Parent and Child Intuitive Eating: A Scoping Review [file sj-pdf-1-ajl-10.1177_15598276241279223.pdf]

Table S1. Search Strategy Sample for MEDLINE performed February 12th, 2024. Childhood BMI was included in the search strategy because it was an outcome included in an earlier draft of the scoping review. The review no longer includes outcomes regarding childhood BMI..

**Concept**	**Search Terms**
Intuitive Eating	1. intuitive eat* or intuitive diet or intuit*<br>2. mindful eat* or mindful* diet or mindful*
Parental	3. matern* or patern* or parent* or famil* or family-based
Childhood BMI	4. child* or pediatric* or adolescen* or youth or young<br>5. obes* or overweight or weight or size or BMI or body mass index
	6. 1 OR 2 (n = 37889)<br>7. 4 AND 5 (n = 478975)
	**14. 3 AND 6 AND 7 (n = 150)**
